# Optimizing Cu electrocatalysis using programmed alternating current

**DOI:** 10.1093/nsr/nwae298

**Published:** 2024-08-23

**Authors:** Jonathan Lei, Song Lin

**Affiliations:** Department of Chemistry and Chemical Biology, Cornell University, USA; Department of Chemistry and Chemical Biology, Cornell University, USA

**Figure 1.**
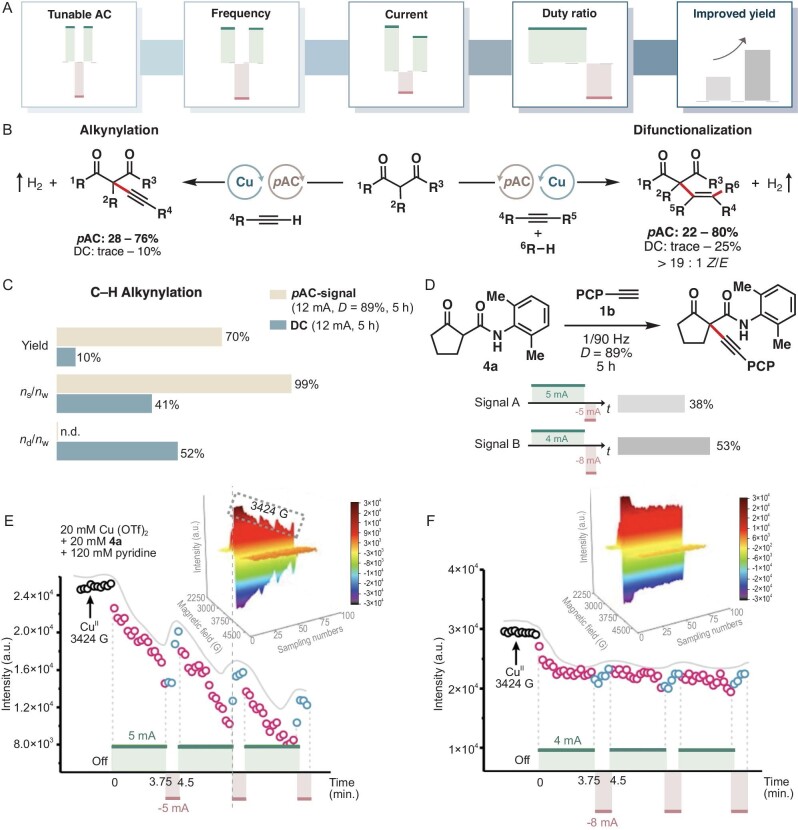
Tunable AC parameters to optimize Cu-catalyzed C–C coupling. (A) AC parameters that can be tuned to improve yield. (B) Two model reactions used in the study: alkynylation and alkyne difunctionalization. (C) Yield and percentage of Cu catalyst deposition in the alkynylation reaction under AC and DC conditions. *D* = duty ratio; *n*_s_/*n*_w_ = ratio of solution-phase catalyst to total catalyst; *n*_d_/*n*_w_ = ratio of deposited catalyst to total catalyst. (D) Current optimization for alkynylation. PCP = para-chlorobenzene. (E) *In-situ* EPR monitoring of active Cu(II) in alkynylation using signal A. (F) *In-situ* EPR monitoring of active Cu(II) in alkynylation using signal B. Panels (E) and (F) are adapted from Ref. [5].

In the past decade, electrochemistry has become widely recognized by the broader chemistry community as an enabling tool for chemical synthesis. One unique feature of electrochemistry is that it allows users to temporally control the reaction by changing the electrical input during electrolysis. This feature, however, has been rarely used in electroorganic synthesis until recently. Alternating current (AC) electrolysis is a technique in which the polarity of the electrodes is periodically inverted, with the anode becoming the cathode and vice versa. In recent years, AC electrolysis has been used to enable previously challenging transformations by affecting sequential reduction and oxidation events [1], modulating local pH near the electrode [2], or switching between single- vs. two-electron redox processes [3]. AC electrolysis also provides a practical solution to avoid reductive deposition of transition metals, promoting catalytic performance [4]. Despite these advances, the potential of AC methods in innovating organic synthesis remains to be fully realized. AC provides a suite of new parameters that could be tuned to accomplish desired functions, including frequency, current, and duty ratio (amount of positive half-period over a whole AC period) (Fig. 1A). These factors can affect when, how, and how fast electron transfer takes place. However, they also complicate the parameter space for reaction optimization.

In a recent report, Lei and coworkers advanced the notion of programmed AC (pAC), where parameters including current, frequencies, and duty ratios can all be tuned to optimize Cu-catalyzed C–C bond coupling reactions (Fig. 1B) [5]. Under direct current (DC) conditions, this type of reaction suffers from catalyst deposition on the counter electrode due to the favorable reduction of Cu ions to metallic Cu [6]. This limitation was cleverly addressed using AC electrolysis, which allowed deposited Cu to be stripped and redissolved into the solution upon polarity reversal from reducing to oxidizing on the cathode. In particular, it was found that by tuning the duty ratio and the current magnitude in the opposite half-periods, maximum yield could be achieved, and the amount of deactivated catalyst at the end of the reaction could be reduced from >50% to <3% (Fig. 1C).

Lei *et al*. performed a series of studies to understand the mechanism basis for the pAC-enabled optimization. They employed *in-situ* electron paramagnetic resonance (EPR) spectroscopy to monitor the concentration of the active Cu(II) catalyst, which shed light on the dependence of catalyst concentration on duty ratio and magnitude of current in each half-period. It was found that during the alkynylation reaction, the Cu(II) concentration decreases during the positive half-period (i.e. when an oxidizing current is applied on the working electrode and a reducing current applied on the counter electrode) and increases during the opposite negative half-period (Fig. 1E). These insights allowed the authors to further tune the currents to maintain a more steady Cu(II) concentration, leading to improved alkynylation yield (Fig. 1D and F).

Under optimal AC conditions, a broad substrate scope was achieved for both target transformations. This workflow encompassing pAC and *in-situ* EPR monitoring will open new opportunities to innovate in organic synthesis using electrochemistry.
